# Guideline adherence for the treatment of advanced schistosomiasis japonica in Hubei, China

**DOI:** 10.1007/s00436-014-4143-y

**Published:** 2014-10-02

**Authors:** Fangying Zhong, Chenxi Liu, Xinping Zhang

**Affiliations:** School of Medicine and Health Management, Tongji Medical College, HuaZhong University of Science and Technology, No. 13 Hangkong Road, Wuhan, Hubei China

**Keywords:** Guideline adherence, Physicians’ compliance, Advanced schistosomiasis japonica

## Abstract

This study compared physicians’ practices on three treatment procedures and hospitalization days with guideline recommendations to assess guideline adherence in the treatment of advanced schistosomiasis japonica. Descriptive statistics were used to estimate patients’ characteristics and rate of guideline adherence. And chi-square tests were used to assess influences of severity of the disease on guideline adherence. The study found no one (0/173) adhered to adequate diagnosis, treatment regimens, and discharge criteria of guidelines completely. And 2.23 % of patients in group 1 and 4.23 % in group 2 were totally conforming to adequate diagnosis. 91.91 % of patients were conforming to adequate treatment regimens among which group 1 and group 2 were 90.32 and 92.25 %, respectively. And one (2.23 %) patient in group 1 and zero (0 %) in group 2 were conforming to discharge criteria of guidelines, and most of the patients left hospital without symptom checks (151/173), liver function and biochemical tests (169/173), and complication checks (91/173). Among 173 inpatients, rate of adequate hospitalization days was 36.42 % (63/173). And chi-square test suggested no significant difference (*P* > 0.05) on guideline adherence in two groups, which implied both of critical and general patients’ treatments should be stressed to comply with guidelines. There existed a large gap between guidelines and practices of the treatment of advanced schistosomiasis japonica.

## Introduction

Clinical practice guidelines have proliferated in recent decades as an attempt to improve the effectiveness and efficiency of health care (McCormack and Loewen 2007). Emphasis on the development and implementation of guidelines has been fueled by research documenting high rates of inappropriate care and wide variations in clinical practice for common health care conditions (McGlynn et al. 2003; Bampton et al. 2007). Adherence to clinical practice guidelines is advocated as a method to decrease utilization of ineffective therapies and increase adoption of evidence-based practices, ultimately resulting in improved patient outcomes and more cost-effective care. Belief in the ability of guidelines to improve quality of care has led to a rapid proliferation in the number of guidelines available for a wide variety of clinical conditions (Larson 2003; Walter et al. 2009; Moonen and Cohe 2011). Although the primary goal of clinical practice guidelines is to improve quality, few of researches have actually examined the impact of adherence to guideline recommendations on clinical diagnosis, treatment regimens, and discharge criteria (Fritz et al. 2007).

Schistosomiasis affects about 74 countries and regions containing 200 million people worldwide, and more than 650 million people live in endemic areas. Several million people all over the world suffer from severe morbidity as a consequence of schistosomiasis. Its infections impose a great burden on poor populations in the developing world (WHO 2006). China is an endemic area of schistosoma japonica and one of the four countries most seriously harmed by schistosomiasis in the globe (Ren 2009; Zhu et al. 2010). Much billion Yuan was expended to control the disease, with the majority of costs related to prevention as well as prescription medication. Like other common conditions, inappropriate variations in care and unacceptably high use of ineffective therapies might lead to serious organic injury or complication such as upper gastrointestinal bleeding (UGB) (Chen 2002). All of the above calls for increased adherence to clinical practice guidelines of schistosomiasis.

Although all patients with schistosomiasis seek care and receive therapy in the local lazaret or the designated hospital and they are managed in local Center for Disease Control and Prevention (CDC), there still exists incorrect and unreliable diagnosis, etc. (Feng et al. 2011). Because of serious consequence of schistosomiasis, many clinical practice guidelines have been developed to direct primary care providers towards more evidence-based management (Lugtenberg 2011). Guidelines provide a series of active criteria for clinical diagnosis and treatment regimens so that passive treatment could be minimized (Hope et al. 2008). Therefore, adherence to guidelines by clinicians managing patients with schistosomiasis has been of great concern.

The principle of diagnosis and treatment guideline is accordant with most patients, so guideline adherence should be promoted (Shao 2012; Fenwick et al. 2009). However, study exploring the adherence to guideline recommendations of schistosomiasis is lacking. Most researches have examined the impact of adherence on costs and process-related variables (Cretin et al. 2007). The study took clinical diagnosis, treatment regimens, and discharge criteria into consideration and analyzed the targets in relation to guideline adherence. And our study, by presetting a research hypothesis, devoted that there was approving guideline adherence in the treatment of patients with advanced schistosomiasis. The main purpose of this study was to examine adherence to the guideline recommendations for the practice provided by physicians to patients with advanced schistosomiasis and finally to promote rational and adequate schistosomiasis care.

## Methods

### The criteria for evaluating guideline adherence

Guidelines for the study reference are the Ministry of Health of the People’s Republic of China (Diagnostic Criteria for Schistosomiasis) and Disease Prevention and Control Department of the Ministry of Health of the People’s Republic of China (Schistosomiasis Control Manual (the third edition)). On the basis of the guidelines above, a criteria was formulated for the adherence study as shown in Table 1 (Schwentner et al. 2013). The criteria contained guideline recommendations of three treatment procedures, which were diagnosis, treatment regimens, and discharge check, respectively. According to the criteria, we did a comparative analysis with the actual treatment for patients with advanced schistosomiasis japonica.

**Table 1:** The criteria for evaluating guideline adherence based on Diagnostic Criteria for Schistosomiasis and Schistosomiasis Control Manual (the third edition)

	Conforming to guideline	Nonconforming to guideline
Diagnosis		
History of infected water contact	Duration of contact with infected water or prior schistosomiasis treatment	Neither duration of contact with infected water nor prior schistosomiasis treatment
Clinical manifestations	Portal hypertension, dwarf or colonic granuloma	Without portal hypertension, dwarf and colonic granuloma
Etiological examination	Find worm eggs or miracidium	Never find worm eggs or miracidium
Immunologic test	Patients without history of schistosomiasis treatment or with more than 2 years of treatment: IHA ≥ 1:10, one or more of ELISA, DIGFA, and DDIA were positive	Patients without history of schistosomiasis treatment or with more than 2 years of treatment: IHA < 1:10, all of ELISA, DIGFA, and DDIA were negative
Treatment regimens		
No praziquantel therapy	Taking	Non-taking
Liver-protecting therapy	Taking hepatinica	Not taking hepatinica
Symptomatic therapy	Done	Not done
Discharge criteria, hospitalization days		
Patient’s state on discharge	Vital signs turning normal	Vital signs not turning normal
Symptom check	No fever, no abdominal pain, etc.	Fever, abdominal pain, etc.
Liver function and biochemical test	Turning normal	Not turning normal
Complications	No severe complications	Severe complications
Hospitalization days	10 ~ 15 days	<10 or >15 days

### Study population

A retrospective study of patients with advanced schistosomiasis japonica was conducted from July 2013 to February 2014. Patients with advanced schistosomiasis at two Chinese representative hospitals including Gong’an Schistosomiasis Special Hospital and Shishou Third People’s Hospital were included in the study and eligible if they (1) were not seriously ill or without any other disorder; (2) do not have serious schistosomiasis complications and organic diseases, hepatitis, or any other liver dysfunction; (3) were over 4 years of age (WHO 2006); (4) were without history of adverse drug reactions; and (5) have complete medical records.

### Data collection

One hundred seventy-three patients’ general characteristics, which contained sex, age, state of health, job, and history of disease, were recorded at admission from January 2010 to November 2013. We would assess the guideline adherence in the treatment for patients with advanced schistosomiasis japonica. So, the related diagnosis, treatment regimens, and discharge check of these patients were collected by rule and line.

Because the severity of disease was one of the most important factors which influenced the practices of physicians’ diagnosis and treatment behavior (Chen 2007; Ma and Zhong 2004), the study divided the 173 patients into two groups. Group 1 comprises 31 critical patients, and group 2 comprises 142 general patients according to severity of advanced schistosomiasis japonica.

### Statistical analysis

Descriptive statistics were used to estimate patients’ characteristics in the study population and to compare the two groups according to their characteristics concordance status. To further elucidate the impact of guideline adherence, we calculated the rate of adequate diagnosis, treatment regimens, and discharge check, respectively, and the total rate of all the three procedures conforming to guideline recommendations as seen in the following formulas. Then, for the above three procedures, chi-square tests were used to assess differences between the two groups, when appropriate. All tests were bilateral, and a *P* value of <0.05 was considered statistically significant. Statistical analyses were performed using Statistical Package for the Social Sciences (SPSS) 13.0. Finally, through comparative analysis, guideline adherence would be stated clearly.$$ \begin{array}{c}\hfill \mathrm{Rate}\ \mathrm{o}\mathrm{f}\ \mathrm{adequate}\kern0.1em A*=\mathrm{Total}\ \mathrm{number}\ \mathrm{o}\mathrm{f}\ \mathrm{adequate}\ A*/\mathrm{Total}\ \mathrm{number}\ \mathrm{o}\mathrm{f}\ A \times 100\ \%\hfill \\ {}\hfill \left(*A\kern0.1em \mathrm{represented}\kern0.1em \mathrm{diagnosis},\kern-0.1em \mathrm{t}\mathrm{reatment}\kern0.58em \mathrm{regimens},\kern0.58em \mathrm{and}\kern0.58em \mathrm{discharge}\kern0.6em \mathrm{check},\kern0.6em \mathrm{respectively}.\right)\hfill \\ {}\hfill \mathrm{Total}\kern0.55em \mathrm{rate}\kern0.55em =\kern0.55em \mathrm{Total}\kern0.5em \mathrm{number}\kern0.4em \mathrm{o}\mathrm{f}\kern0.55em \mathrm{all}\kern0.5em \mathrm{t}\mathrm{he}\kern0.55em \mathrm{t}\mathrm{hree}\kern0.5em \mathrm{procedures}\kern0.2em \mathrm{conforming}\kern0.15em \mathrm{t}\mathrm{o}\kern0.15em \mathrm{guidelines}/\mathrm{Total}\ \mathrm{number}\ \mathrm{in}\ \mathrm{t}\mathrm{he}\ \mathrm{study}\kern-0.1em \times \kern-0.1em 100\kern0.05em \%\hfill \end{array} $$

## Results

### General characteristics

A total of 173 patients with advanced schistosomiasis japonica were studied, 106 of whom were males. Thirty-one patients were included in group 1, and the others were in group 2 (Table 2). There was no statistical significance in terms of sex, age, state of health, job, and history of disease of the patients between group 1 and group 2. Thus, all of the above analysis demonstrated that there was good comparability in the two groups.

**Table 2:** The characteristics of the study groups based on 31 critical patients and 142 general patients with advanced schistosomiasis japonica included in the study

		Group 1	Group 2	*χ* ^2^	*P* value
Sex	Male	15	91	2.642	0.104 *P*
	Female	16	51		
Age	40≤	0	13	5.388	0.068 *P*
	40–60	12	69		
	≥60	19	60		
State of health	Good	2	9	0.089	0.956 *P*
	General	24	113		
	Poor	5	20		
Job	Farmer	30	140	0.428	0.681 *F*
	Else	1	2		
History of disease	No	0	15	3.586	0.058 *P*
	Yes	31	127		

*F* Fisher’s exact test, *P* Pearson’s chi-square test

### Overall guideline adherence

The study found that only one (2.23 %) patient in group 1 was totally in accordance with adequate diagnosis or discharge criteria. And 28 (90.32 %) patients’ treatment regimens were adequate in the group. Besides, there were 6 (4.23 %), 131 (92.25 %), and 0 (0 %) patients, respectively, that conform to adequate diagnosis, treatment regimens, and discharge criteria. What is more, no one (0/173) was conforming to adequate diagnosis, treatment regimens, and discharge criteria according to guidelines at the same time (Table 3).

**Table 3:** The rate of adequate procedure according to guidelines

Adequate procedure	Group 1	Rate^a^ (%)	Group 2	Rate (%)	Total rate (%)
Diagnosis	1	2.23	6	4.23	0 (0/173)
Treatment regimens	28	90.32	131	92.25	
Discharge criteria	1	2.23	0	0	

^a^Rate represents the rate of adequate procedure

### Adherence of diagnosis

Table 4 showed the distribution of four diagnosis practices according to guidelines in the two groups. The rate of adequate recommendation to check history of infected water contact was the highest, which of two groups were 96.77 and 95.77 %, separately. Physicians’ compliance with diagnosis recommendations for clinical manifestation check and etiological examination was on the low side. As for immunologic test, the study found the rate is 70.97 % in group 1 and 65.49 % in group 2. Finally, the chi-square test suggested there was no significant difference (*P* > 0.05) on guideline adherence of the four diagnoses in the two groups.

**Table 4:** Guideline adherence of diagnosis recommendations

		Group 1	Rate (%)	Group 2	Rate (%)	*χ* ^2^	*P* value
History of infected water contact	Yes	30	96.77	136	95.77	0.065	0.798
	No	1		6			
Clinical manifestations	Yes	6	19.35	28	19.72	0.002	0.963
	No	25		114			
Etiological examination	Yes	11	35.48	42	29.58	0.418	0.518
	No	20		100			
Immunologic test	Yes	22	70.97	93	65.49	0.342	0.559
	No	9		49			

### Adherence of treatment regimens

Through statistics, there was a high rate of adequate treatment regimens. In group 1, 100 % treatment regimens of liver-protecting therapy and no praziquantel therapy were conforming to guidelines, and 28 (90.32 %) regimens of symptomatic therapy complied with the guidelines. As for group 2, the rate of three treatment regimens in accordance with guidelines was 99.30, 100, and 92.96 %, respectively. Besides, the chi-square test showed there was no significant difference (*P* = 0.614) on symptomatic therapy in the two groups (Table 5).

**Table 5:** Guideline adherence of treatment regimen recommendations

		Group 1	Rate (%)	Group 2	Rate (%)	*χ* ^2^	*P* value
No praziquantel therapy	Yes	31	100	141	99.30	–^a^	–
	No	0		1			
Liver-protecting therapy	Yes	31	100	142	100	–	–
	No	0		0			
Symptomatic therapy	Yes	28	90.32	132	92.96	0.254	0.614
	No	3		10			

^a^The subjects’ distribution of no praziquantel therapy and liver-protecting therapy in two groups did not meet the criteria of chi-square test

### Adherence of discharge criteria and hospitalization days

Table 6 showed the compliance with guidelines to check patient’s state on discharge was good, as the rate was 96.77 and 97.89 % in the two groups. In contrast, the rate of adequate symptom check and liver function and biochemical test was low. Discharge checks of 13 (41.94 %) patients in group 1 and 69 (48.59 %) in group 2 were conforming to guidelines to check complications. Besides, the chi-square test showed there was no significant difference (*P* > 0.05) on guideline adherence of the four discharge checks in the two groups.

**Table 6:** Guideline adherence of discharge criteria recommendations

		Group 1	Rate (%)	Group 2	Rate (%)	*χ* ^2^	*P* value
Patient’s state on discharge	Yes	30	96.77	139	97.89	0.14	0.550 *F*
	No	1		3			
Symptom check	Yes	4	12.90	18	12.77	0.001	0.973
	No	27		124			
Liver function and biochemical test	Yes	0	0	4	2.82	0.894	0.344 *F*
	No	31		138			
Complications	Yes	13	41.94	69	48.59	0.452	0.501
	No	18		73			

*F* Fisher’s exact test

Among the 173 inpatients, the rate of adequate hospitalization days was 36.42 % (63/173). The other inpatients received the hospitalization care more than 15 days or less than 10 days. The rate of group 1 and group 2 was 29.03 % (9/31) and 38.03 % (54/142) separately, and there was no significant difference (0.346) between the two through chi-square test.

## Discussion

Guideline recommendations may be based on either scientific evidence or the expertise of the guideline developers via a consensus decision-making process (Marciano et al. 2014). In order to cure patients with advanced schistosomiasis japonica better, authorized guidelines should be adhered by physicians to standardize treatment procedures. The primary objective of this study was to assess concordance based on the guideline recommendations for the practice provided by physicians to patients with advanced schistosomiasis.

The study found that no one (0/173) adhered to adequate diagnosis, treatment regimens, and discharge criteria according to guidelines at the same time. And only 2.23 % patients in group 1 and 4.23 % ones in group 2 were totally conforming to adequate diagnosis. However, the above nonstandard behaviors of diagnosis practice would be reasons for misdiagnosis (Chen 2007). Previous researches had found that misdiagnosis could and did occur and was reasonably common with error rates ranging from 1.4 % to a high 20–40 % misdiagnosis rate, and surveys of patients also indicated the chance of experiencing a misdiagnosis to range from 8 to 40 % (Right Diagnosis 2014; Reed and May 2011). Besides, a high misdiagnosis rate of schistosomiasis was shown in China because of nonstandard diagnosis procedures (Wu and Huang 2006; Zhu et al. 2010). There were various reasons as to why a misdiagnosis could occur including errors by physicians. For example, physicians in non-endemic areas were usually unfamiliar with schistosomiasis (Nguyen et al. 2012), and hence, misdiagnosis might occur (Huang and Manderson 2005). Therefore, it was very necessary for physicians to conform to guidelines to diagnose patients with advanced schistosomiasis japonica. In the study, physicians’ compliance with diagnosis recommendations for clinical manifestation check and etiological examination was especially on the low side so that the two diagnosis recommendations should be conformed emphatically.

In the study, there were totally 91.91 % of adequate treatment regimens. And just 13 (7.51 %) regimens of taking symptomatic therapy were not following guidelines, among which there are 3 (3/31) in group 1 and 10 (10/142) in group 2. Research reported that less than 40 % of patients in the public sector and 30 % in the private sector are treated according to clinical guidelines in developing countries (WHO 2010). And these clinical practices might result from irrational drug use. WHO once estimated that more than half of all medicines are prescribed, dispensed, or sold inappropriately and that half of all patients fail to take them correctly (WHO 2014; Fromer and Cooper 2008). Medically inappropriate, ineffective, and economically inefficient use of pharmaceuticals is commonly observed in the health care system throughout the world (Editorial 2012). Researches have shown that irrational treatment could lead to praziquantel-resistant isolates of schistosomiasis (Wang et al. 2012; Cioli and Pica-Mattoccia 2003). Hence, it is crucial to improve physicians’ compliance with treatment regimen recommendations of guidelines, especially in symptomatic therapy (Rumsfel 2011).

All patients with advanced schistosomiasis japonica required hospitalizations. If hospitalization days were not enough, patients would underutilize adequate treatment. And complete discharge checks would make sure whether patients leave hospital appropriately. The study found that only one (2.23 %) patient in group 1 and zero (0 %) in group 2 were in accordance with discharge criteria of guidelines, and most of the patients left hospital without symptom check (22/173), liver function and biochemical tests (4/173), and complication check (82/173). Among the 173 inpatients, the rate of adequate hospitalization days was just 36.42 % (63/173). Besides, previous studies in China have shown that the discharge checks and the hospitalization days were the main factors influencing inpatients’ medical expenses and overuse (WHO 2010; Wang et al. 2009; Gurarie et al. 2010). Therefore, following discharge criteria, especially in the above three aspects of discharge check, and hospitalization of guideline recommendations could effectively lead to rational drug use, adequately treat diseases, and minimize medical service cost.

What is more, the chi-square test suggested that there was no significant difference (*P* > 0.05) on guideline adherence in the two groups in the study. The severity of advanced schistosomiasis japonica could not influence physicians’ practices to adhere to guidelines. Thus, both of critical and general patients’ treatments should be stressed to comply with guidelines.

To our knowledge, this is the first study to evaluate guideline adherence in the treatment for patients with advanced schistosomiasis japonica in the two groups’ clinical practices. Strengths of the study include multiangle analysis of treatment procedures and the disease severity and some important application values for health sectors and policymakers. The findings of the study are subject to some limitations. Because of limited data, we did not study whether these guidelines were adequate or valid for advanced schistosomiasis treatment. Consequently, we assessed compliance based on the guideline recommendations only and did not study the subject in more depth (Richard-Tremblay et al. 2012).

## Conclusions

The large gap between guidelines and the practices of treatment for patients with advanced schistosomiasis japonica was found in the study. Therefore, it is time for health sectors to take measures to standardize physicians’ practice through guidelines.

## Ethics approval

The study was reviewed and approved by the Research Ethics Committee of Tongji Medical College, Huazhong University of Science and Technology (IORG No: IORG0003571).
